# Trends and Factors Associated with Comprehensive Knowledge about HIV among Women in Vietnam

**DOI:** 10.3390/tropicalmed5020091

**Published:** 2020-06-03

**Authors:** Nguyen Van Son, Hoang Duc Luan, Ho Xuan Tuan, Le Manh Cuong, Nguyen Thi Thuy Duong, Vu Duy Kien

**Affiliations:** 1 Phu Tho Provincial General Hospital, Viet Tri, Phu Tho 290000, Vietnam; 2 Phu Tho College of Medicine and Pharmacy, Viet Tri, Phu Tho 290000, Vietnam; 3 School of Medicine and Pharmacy, The University of Da Nang, Da Nang 550000, Vietnam; hxtuan@smp.udn.vn or; 4 National Hospital of Traditional Medicine, Hanoi 100000, Vietnam; cuonglm@nhtm.gov.vn or; 5 National Institute of Hygiene and Epidemiology, Hanoi 100000, Vietnam; nttd@nihe.org.vn or; 6 OnCare Medical Technology Company Limited, Ha Noi 100000, Vietnam; vuduykien@oncare.vn or

**Keywords:** trends, comprehensive knowledge, factors, HIV, women, Vietnam

## Abstract

This study aimed to assess the trends and associated factors of comprehensive knowledge about HIV among women in Vietnam using the dataset of the Vietnam Multiple Indicator Cluster Surveys (MICSs) in 2000, 2006, 2011, and 2014. The outcome variable was comprehensive knowledge about HIV, defined as the ability to correctly answer three knowledge questions and to reject the three most common misconceptions about HIV prevention. We found that comprehensive knowledge about HIV increased from 26.1% in 2000 to 44.1% in 2011, but it decreased slightly between 2011 and 2014, from 44.1% to 42.4%. Increased comprehensive knowledge about HIV was associated with women who had higher education and those in the fourth and fifth quintiles of household wealth in all four rounds of the MICS. Comprehensive knowledge about HIV among women was also associated with those who had ever been tested for HIV and those with knowledge of where to be tested for HIV. Women in the urban areas were more likely to have higher levels of comprehensive knowledge about HIV as compared to the women in the rural areas in 2000, 2006, and 2011 but not in 2014. Comprehensive knowledge about HIV among women in Vietnam increased from 2000 to 2014, but it was still relatively low.

## 1. Introduction

Comprehensive knowledge about human immunodeficiency virus (HIV) includes an understanding of HIV transmission routes and the ability to counter local misconceptions about HIV/AIDS. The definition of comprehensive knowledge of HIV may vary slightly between studies or countries but is considered here to be the comprehensive knowledge of HIV of an individual. According to an estimation by UNAIDS, new cases of HIV decreased markedly from a peak of 2.9 million people in 1997 to 1.7 million people in 2018 [1]. Although there are global plans and strategies towards ending the HIV pandemic, it is unlikely for this to happen by 2030 [2,3,4]. The prevalence of HIV in high-risk groups is still high, and there is a potential risk of its spread among communities if preventive measures are not maintained and strengthened [1]. Strengthening comprehensive knowledge about HIV is considered to be an effective solution to prevent HIV in countries around the world [5,6]. Furthermore, whereas women are more likely to acquire HIV than men [7,8], the global prevalence of comprehensive knowledge about HIV is still lower among women than men [6].

While the HIV epidemic in Vietnam is still considered to be in the "concentrated stage" because the current HIV prevalence rate is less than 1%, prevalence is higher among high-risk groups, such as injected drug-users and female sex workers [9,10]. Though about 230,000 people are living with HIV in Vietnam, the number of new cases decreased sharply from an estimated 16,000 people in 2010 to 5700 people in 2018 [1]. To cope with the HIV epidemic, Vietnam is implementing interventions focusing primarily on groups with a high risk of contracting HIV and is also developing programs to improve public knowledge about HIV [11]. In addition to government efforts, international organizations have also contributed significantly to the fight against the HIV epidemic in Vietnam. However, after 2010, when Vietnam progressed from a low-income country to a lower middle-income country, international resources for HIV prevention in Vietnam decreased substantially [12]. Although Vietnam made certain policy reforms to mobilize domestic resources, the budget gap from international organizations affected HIV prevention program outcomes [11]. The level of knowledge of HIV and its prevention and control was low and not regularly assessed in Vietnam [11]. In the most recent study, Huy et al. investigated HIV knowledge over a period of time, although only data collected before 2011 were included [13]. Another study in Vietnam investigated knowledge of HIV transmission from mothers to children [14]. To our knowledge, no studies using any definition of comprehensive knowledge about HIV have been conducted in Vietnam. As such, we conducted this study due to this gap in the literature and in order to investigate the state of comprehensive knowledge about HIV among women. Therefore, the aim of this study was to assess the trends and associated factors of comprehensive knowledge about HIV among women in Vietnam.

## 2. Methods

### 2.1. Sources of Data

This study was a secondary analysis of the Vietnam Multiple Indicator Cluster Surveys (MICSs) data from 2000, 2006, 2011, and 2014 [15,16,17,18]. The first round of MICS was conducted in 1996, but its questionnaires did not include information about HIV. Thus, we did not include the data in 1996 for analysis. The global MICS program was initiated by the United Nations Children’s Fund (UNICEF) to help countries around the world to monitor indicators related to women and children. With the technical and financial support of UNICEF, the MICSs in Vietnam were conducted by the General Statistics Office of Vietnam. The MICSs were nationally-representative household surveys. In Vietnam, five MICSs were conducted every four to six years beginning in 1996, of which the fifth and most recent was conducted in 2014. In each MICS, two-stage sampling was performed by region and then stratified for area type (urban or rural). Cluster numbers per region were calculated according to sample size estimations, and each cluster (primary sampling unit) included 20 households. The probability proportional to the size sampled was applied to select clusters in each region. Within each cluster, the random systematic selection was used to select 20 households. The estimation of the sample size and sampling method has been described in detail elsewhere [15,16,17,18]. In this study, we extracted data for women aged 15–49 years old. The number of women with complete information included in the analysis was 9252 in 2000, 9471 in 2006, 11,663 in 2011, and 9827 in 2014.

### 2.2. Variables

The outcome variable was considered to be binary as determined by whether a woman had comprehensive knowledge about HIV, defined here as the ability to correctly answer all three knowledge questions and to reject all three most common misconceptions about HIV prevention. The three knowledge questions included the consistent use of condoms during sexual intercourse, that having a single uninfected and faithful partner can reduce the chance of contracting HIV, and that a healthy-looking person can carry HIV. The three local misconceptions about HIV included transmission through mosquito bites, transmission via supernatural means, and transmission via sharing food with a person infected with HIV. The independent variables in this study included age group (<20, 20–34, or 35–49 years), marital status (single or currently married), place of residence (urban or rural), ethnic group (Kinh/Hoa or ethnic minority), and education level (primary or lower, lower secondary, upper secondary, or tertiary). We considered two other variables related to the experiences of women concerning HIV, including having ever been tested for HIV status (yes or no) and knowing where to be tested for HIV (yes or no). In this study, socioeconomic status was categorized by five equal quintiles, including the poorest, poor, middle, rich, and richest groups. The variable of socioeconomic status was estimated and provided in the dataset [15,16,17,18]. Basically, the socioeconomic status was determined based on the wealth asset index. To calculate the wealth asset index, principal component analysis (PCA) was performed by using variables of valuable household goods, such as electricity, radios, televisions, telephones, refrigerators, beds, tables, sofas, fans, computers, air-conditioners, gas cookers, electric cookers, electric cookers, washing machines, tractors, cars or ships, and boats with motors. In addition, other variables might be included in the PCA model, such as household characteristics and the status of water and sanitation. After analysis, each household was assigned a wealth asset score, and based on that, the households were divided into five equal quintiles. The detailed method to estimate the wealth asset index was described in the MICS reports [15,16,17,18].

### 2.3. Statistical Analysis

Descriptive analysis was used to estimate the percentage of comprehensive knowledge concerning HIV among women in each survey round. We also calculated the percentage and 95% confidence interval (CI) of comprehensive knowledge about HIV among women according to various factors including age group, marital status, place of residence, ethnicity, educational attainment, household socioeconomic status, having been tested for HIV, and knowing where to be tested for HIV. Changes in the percentage of comprehensive knowledge about HIV among women between 2000 and 2014 were also estimated according to various factors. We conducted a multivariate logistic regression analysis to identify factors associated with comprehensive knowledge about HIV among women. All the statistical analyses in this study were conducted using STATA® 14.0 (StataCorp, College Station, Texas, USA) using the weighting of variables for women in the dataset. Differences were considered significant at p-values less than 0.05.

## 3. Results

**Figure 1:**
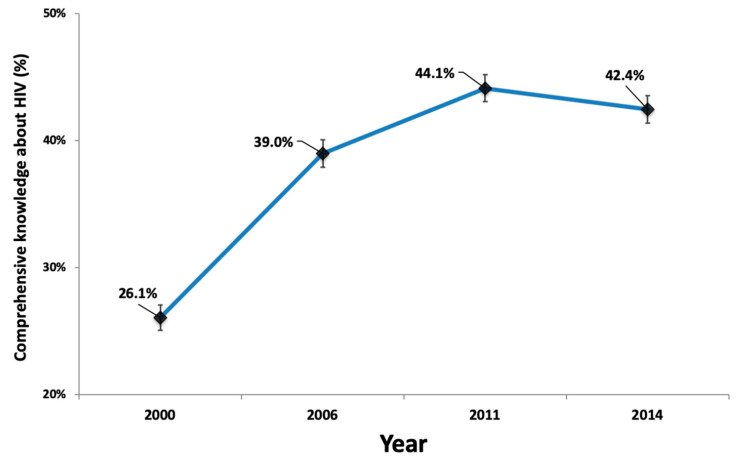
Trends in the percentage and 95% confidence intervals of comprehensive knowledge of HIV among women aged 15–49 years in Vietnam (weighted), 2000–2014.

Table 1 presents the weighted characteristics of the women aged 15–49 years in the study according to various factors from 2000 to 2014. Age group, marriage status, ethnicity, and socioeconomic status structures were relatively similar among the MICS surveys. The proportion of women in urban areas increased from 25.8% in 2000 to 33.2% in 2014. The proportion of women with upper secondary and tertiary educational attainment increased, while the proportion of women with less education (primary or lower secondary) tended to decrease over the study period. The proportion of women who had ever been tested for HIV increased from 5.6% in 2000 to 21.5% in 2014. The percentage of women with knowledge of where to be tested for HIV increased sharply from 24.2% in 2000 to 59.1% in 2006, decreased to 38.8% in 2011, and increased slightly to 40.9% in 2014. Trends in the percentage and 95% CIs (weighted) of comprehensive knowledge about HIV among women aged 15–49 years estimated in the different rounds of the MICS from 2000 to 2014 are shown in Figure 1. The percentage of comprehensive knowledge about HIV among women aged 15–49 years increased sharply from 26.1% in 2000 to 44.1% in 2011 but then decreased slightly to 42.4% in 2014.

Table 2 shows the weighted percentage and 95% CI of comprehensive knowledge about HIV among women aged 15–49 years according to various factors from 2000 to 2014. The percentage of comprehensive knowledge about HIV among women according to various factors increased sharply between 2000 and 2011. However, this proportion decreased slightly between 2011 and 2014 for each factor. Younger women tended to have comprehensive knowledge about HIV at a higher rate than did than older women, whereas single women had a higher rate of comprehensive knowledge about HIV than did married women. Women living in urban areas, those of the Kinh and Hoa ethnic groups, those with higher education, and wealthier women had higher levels of comprehensive knowledge about HIV as compared to women living in rural areas, those belonging to minority ethnic groups, those having less formal education, and those with less wealth. Women who had ever been tested for HIV had a higher level of comprehensive knowledge about HIV than did women who had never been tested for HIV. Except for the MICS conducted in 2000, women who knew where to be tested for HIV had a higher level of comprehensive knowledge about HIV than did women who did not. In addition, Table 2 shows the percentage change in comprehensive knowledge about HIV among women between the years 2000 and 2014. The change was higher among women aged less than 20 years, single women, inhabitants of rural areas, those who belonged to the Kinh and Hoa ethnic groups, those with higher education, wealthier women, those who had ever been tested for HIV, and those with knowledge of where to be tested for HIV.

Table 3 presents associations between various factors with comprehensive knowledge about HIV among women according to multivariate logistic regression analysis. Increased comprehensive knowledge about HIV was associated with women who had higher education and those in the fourth and fifth quintiles of household wealth in all four rounds of MICS. Except for study participants in the MICS from the year 2000, increased levels of comprehensive knowledge about HIV among women were associated with those who had ever been tested for HIV and those with knowledge of where to be tested for HIV. Women in the urban areas were more likely to have higher levels of comprehensive knowledge about HIV as compared to the women in the rural areas in 2000, 2006, and 2011 but not in 2014. The women in the 20–34 age group had increased levels of comprehensive knowledge about HIV only in 2011 and 2014.

## 4. Discussion

Our study indicates that the percentage of comprehensive knowledge about HIV among women in Vietnam increased significantly from 2000 to 2011, but then decreased slightly between 2011 and 2014. The relationship between comprehensive knowledge about HIV with various factors differed between rounds of the MICS. However, women with higher levels of education and those belonging to wealthier households had consistently higher levels of comprehensive knowledge about HIV as compared to those with primary or lower educational attainment and those belonging to the poorest households. In addition, having ever been tested for HIV along with knowledge of where to be tested for HIV were both associated with higher levels of comprehensive knowledge about HIV. The relatively low level of comprehensive knowledge about HIV should be given more attention by health policymakers, especially given the reduction in knowledge that occurred between 2011 and 2014. With limited resources, the determination of factors that are associated with comprehensive knowledge about HIV may help to guide appropriate interventions in the future.

Although the percentage of comprehensive knowledge of HIV among women in our study tended to increase during the study period, it was still less than 50% in 2014. Our results are similar to those of other studies that reported the percentage of comprehensive knowledge about HIV among women [5,19,20,21,22]. Despite the efforts of many global intervention projects, the percentage of comprehensive knowledge about HIV among women was still low [9]. One study in Kenya indicated that the percentage of comprehensive knowledge about HIV among women was only 9% in 1993 but that it increased to 54% in 2009 [23]. Although the definition of comprehensive knowledge about HIV among women differed from that used in our study, Huy et al. also reported that the percentage of HIV knowledge among women in Vietnam increased from 23.7% to 44.7% between 2000 and 2001 [13]. In addition, another study in Vietnam also suggested that the percentage of women who knew about measures used to prevent the mother-to-child transmission of HIV was still low and ranged from 41.8% in 2000 to 46.8% in 2014 [14].

The Vietnamese government has focused on public knowledge of HIV, as is clearly reflected in the law on HIV/AIDS prevention and control [24]. An important topic that Vietnam has prioritized is the dissemination of information, education, and communication of HIV/AIDS prevention and control knowledge. In addition, many measures designed to combat the HIV epidemic in Vietnam have been implemented and have been effective in reducing the incidence of new cases and increasing the proportion of cases treated with anti-retroviral (ARV) drugs [10]. However, international resources for HIV/AIDS prevention and control have decreased sharply since Vietnam became a middle-income country in 2010 [11]. Furthermore, the mobilization of domestic resources was still limited, leading to potential negative impacts on HIV/AIDS prevention programs in Vietnam [11]. Typically, when resources are limited to activities that focus on the treatment and procurement of ARV therapies, the resources available for communication and education are likely reduced. However, communication and education are required to improve public knowledge about HIV, including that of women. Thus, we believe that health care policymakers in Vietnam should give additional attention to communication and education activities in order to improve comprehensive knowledge of HIV among women. In particular, it is necessary to develop a specific roadmap to achieve 80% comprehensive knowledge about HIV as recommended by UNAIDS [3,7].

We have found that the level of educational attainment was closely related to comprehensive knowledge about HIV through all the survey rounds. This result was consistent with a number of previous studies in Vietnam and around the world [13,21,22,23]. With limited resources, it is essential to clearly identify target groups for interventions. Of these, people with less formal education may require prioritization from HIV prevention activities. Meanwhile, education and communication regarding HIV must continue to be expanded to highly educated people, who may then be selected to participate in further community education activities. In Vietnam, the Vietnamese Women’s Union is a broad community network that could be the basis for strengthening awareness and intervention campaigns [25].

We found that wealthier women had a higher level of comprehensive knowledge about HIV than did poorer women. These results were consistent across the surveys in 2000, 2006, and 2014. In 2011, we only found a significant difference between the richest and poorest quintiles. This could be explained by the fact that HIV prevention interventions were promoted in the previous period, in which groups of women in the community raised awareness about HIV. The survey results also showed an increase in the percentage of comprehensive knowledge about HIV between 2000 and 2011. However, the difference in the level of comprehensive knowledge about HIV among women occurred again in women from the wealthiest households as compared to women from the poorest households in 2014. It may be that the decline in intervention activities may have affected HIV prevention in the past [11]. Women from wealthier households could be expected to have more opportunities to access HIV prevention knowledge than those from poorer households. Our findings are similar to those of other studies in Vietnam and other countries [13,20,23]. Therefore, we believe that women from poorer households should be prioritized in HIV prevention interventions.

A few previous studies have reported significant differences in the levels of comprehensive knowledge about HIV among women when comparing women from urban and rural areas [13,26]. Specifically, people living in urban areas were more likely to have comprehensive knowledge about HIV than were people living in rural areas. In our study, we noted such differences in 2000, 2006, and 2011 but not in 2014. It is possible that past efforts towards HIV prevention interventions had a positive effect on women living in rural areas. We also found that the percentage of comprehensive knowledge about HIV among women living in rural areas was significantly higher (17.2% increase between 2000 and 2014) than that of women living in the urban areas (9.7% increase between 2000 and 2014). However, in the most recent survey round (2014), the percentage of comprehensive knowledge about HIV among women living in rural areas was still much lower than that among women living in urban areas. Therefore, awareness efforts regarding HIV prevention should potentially prioritize women living in rural areas. In addition, we saw no difference in comprehensive knowledge about HIV among women belonging to the Kinh and Hoa ethnic groups and those belonging to minority ethnic groups in the multivariate analysis for the most recent survey round (2014). Even so, ethnic minority women should still be prioritized by HIV prevention interventions because the percentage of comprehensive knowledge about HIV among ethnic minority women (25.1%) was much lower than that of the women belonging to the Kinh/Hoa ethnic groups (45.3%).

Women who had ever been tested for HIV had increased comprehensive knowledge about HIV as compared to those who had not, demonstrating that women who had been tested were able to gain knowledge about HIV prevention when using health care services or HIV counseling services [13,27,28]. In Vietnam, late-term pregnant women are tested for HIV and may be educated regarding HIV transmission prevention during prenatal follow-ups. In addition, we found that a high level of comprehensive knowledge about HIV was associated with knowing where to be tested for HIV. For the prevention of HIV, the availability of medical services that meet diagnosis and prevention requirements is essential. However, if communication of the existence of HIV-related services is insufficient, people may be prevented from being able to access such services. Our research findings indicate that women might receive information through communication activities such as HIV counseling and testing services, echoing a previous study that also documented a relationship between knowing where to be tested for HIV and knowledge about HIV [13]. When women have access to HIV counseling and testing services, additional knowledge about HIV may be provided. Therefore, ensuring the widespread presence of HIV counseling and testing services is essential for HIV prevention in Vietnam.

Our study has several limitations that need to be considered when interpreting the results. The data source for this study was based on self-reported information from respondents, suggesting that the data may be subject to recall bias. The proportion of comprehensive knowledge about HIV was also estimated from self-reported information and could be underestimated or overestimated. Using local misconceptions about HIV to construct comprehensive knowledge about HIV might make it difficult to compare results between regions. Although the MICSs were designed to be nationally representative, sample frame variations between the survey rounds may also influence the estimates in this study. While non-response adjustment factors were included in the sample size calculation, the MICS dataset does not include non-response rate information, which could impact the results of this study. The most recent MICS conducted in Vietnam was completed in 2014, so any more-recent changes cannot be assessed. Finally, given that the MICS surveys were cross-sectional, causal relationships cannot be discovered.

## 5. Conclusions

Our study found that comprehensive knowledge about HIV among women in Vietnam increased from 2000 to 2014 but was still relatively low. As such, appropriate interventions should be implemented in order to increase the proportion of women who possess comprehensive knowledge about HIV. Among these, interventions should especially prioritize women with less formal education and those that belong to the poorest households. Finally, further studies should be conducted in order to uncover additional factors that may impact comprehensive knowledge of HIV among women.

## Figures and Tables

**Table 1:** Characteristics of women aged 15–49 years in the study according to various factors (weighted), 2000–2014.

Chracteristics	Percentage			
	Year 2000	Year 2006	Year 2011	Year 2014
Age group (years)				
<20	24.0	21.3	17.5	16.7
20–34	39.7	36.8	42.0	40.3
35–49	36.3	41.9	40.5	43.0
Marital status				
Single	31.9	30.4	28.5	28.4
Currently married	68.2	69.6	71.5	71.6
Area				
Rural	74.2	73.5	68.5	66.8
Urban	25.8	26.6	31.5	33.2
Ethnicity				
Ethnic minority	12.8	15.2	12.1	14.0
Kinh/Hoa	87.3	84.9	87.9	86.1
Education				
Primary or less	32.3	48.8	20.4	19.9
Lower secondary	42.2	35.0	38.7	35.8
Upper secondary and tertiary	25.5	16.2	40.9	44.3
Socioeconomic status (quintile)				
Poorest 20%	17.9	14.7	17.7	18.0
Near poor 20%	19.4	16.9	18.9	18.5
Middle 20%	20.1	20.7	20.8	20.4
Richer 20%	20.3	22.7	21.3	22.1
Richest 20%	22.3	25.0	21.4	21.0
Ever tested for HIV				
No	94.4	92.3	83.2	78.5
Yes	5.6	7.7	16.8	21.5
Knows where to be tested for HIV				
No	75.8	40.9	61.2	59.2
Yes	24.2	59.1	38.8	40.9

**Table 2:** Percentage and 95% CI of comprehensive knowledge about HIV among women aged 15–49 years according to various factors (weighted), 2000–2014.

Chracteristics	Year 2000 % [95% CI]	Year 2006 % [95% CI]	Year 2011 % [95% CI]	Year 2014 % [95% CI]	Change between 2000 and 2014 % [95% CI]
Age group (years)					
<20	26.5 [24.5, 28.6]	44.8 [42.4, 47.1]	50.3 [47.8, 52.9]	48.9 [46.2, 51.5]	22.4 [18.9, 25.7]
20–34	25.2 [23.7, 26.8]	40.2 [38.5, 42.1]	47.7 [46.0, 49.3]	46.9 [45.2, 48.6]	21.6 [19.3, 24.0]
35–49	26.6 [25.1, 28.4]	34.9 [33.3, 36.6]	37.7 [36.2, 39.3]	35.8 [34.2, 37.4]	9.1 [6.8, 11.4]
Marital status					
Single	28.1 [26.4, 29.9]	47.4 [45.4, 49.5]	51.2 [49.3, 53.2]	50.1 [48.0, 52.1]	21.9 [19.2, 24.7]
Currently married	25.1 [23.9, 26.3]	35.3 [33.9, 36.6]	41.3 [40.0, 42.5]	39.4 [38.2, 40.7]	14.3 [12.6, 16.1]
Area					
Rural	20.3 [19.2, 21.4]	33.3 [32.1, 34.5]	38.1 [36.7, 39.4]	37.5 [36.1, 38.9]	17.2 [15.4, 18.9]
Urban	42.8 [40.7, 45.0]	54.7 [52.5, 56.8]	57.2 [55.7, 58.8]	52.5 [50.8, 54.2]	9.7 [6.9, 12.4]
Ethnicity					
Ethnic minority	13.7 [11.8, 15.8]	30.5 [28.2, 32.9]	28.3 [25.8, 30.9]	25.1 [22.9, 27.4]	11.4 [8.4, 14.4]
Kinh/Hoa	27.9 [26.8, 29.0]	40.5 [39.3, 41.7]	46.3 [45.2, 47.4]	45.3 [44.1, 46.5]	17.4 [15.7, 19.0]
Education					
Primary or less	10.5 [9.4, 11.8]	28.8 [27.4, 30.3]	19.4 [17.7, 21.3]	15.6 [13.9, 17.5]	5.1 [2.9, 7.3]
Lower secondary	24.7 [23.2, 26.2]	42.8 [40.9, 44.7]	37.3 [35.6, 38.9]	34.4 [32.7, 36.2]	9.8 [7.4, 12.1]
Upper secondary and tertiary	48.1 [45.9, 50.3]	61.3 [58.6, 63.9]	62.9 [61.3, 64.5]	60.9 [59.4, 62.6]	12.9 [10.1, 15.6]
Socioeconomic status (quintile)					
Poorest 20%	8.4 [7.1, 10.0]	22.9 [20.7, 25.2]	27.7 [25.4, 30.1]	20.7 [18.7, 22.8]	12.2 [9.7, 14.7]
Near poor 20%	16.1 [14.3, 18.0]	30.1 [27.8, 32.6]	35.6 [33.2, 38.1]	34.7 [32.2, 37.3]	18.7 [15.5, 21.8]
Middle 20%	25.4 [23.2, 27.7]	32.2 [29.9, 34.6]	40.1 [37.7, 42.5]	39.6 [37.2, 42.1]	14.2 [10.9, 17.6]
Richer 20%	31.9 [29.5, 34.3]	41.9 [39.5, 44.3]	46.8 [44.6, 49.1]	48.9 [46.6, 51.3]	17.1 [13.7, 20.4]
Richest 20%	44.4 [42.0, 46.8]	57.3 [55.0, 59.5]	66.5 [64.5, 68.4]	63.8 [61.6, 66.0]	19.4 [16.2, 22.7]
Ever tested for HIV					
No	25.4 [24.4, 26.5]	38.2 [37.1, 39.3]	41.8 [40.7, 42.9]	39.3 [38.1, 40.6]	13.9 [12.3, 15.5]
Yes	36.7 [32.3, 41.5]	48.5 [44.3, 52.6]	55.5 [52.9, 58.1]	53.8 [51.4, 56.1]	17.0 [11.9, 22.2]
Knows where to be tested for HIV					
No	26.9 [25.9, 28.2]	27.5 [25.9, 29.1]	37.8 [36.4, 39.1]	36.5 [35.2, 37.9]	9.6 [7.8, 11.4]
Yes	23.2 [21.3, 25.2]	46.9 [45.5, 48.4]	54.1 [52.4, 55.8]	50.9 [49.2, 52.7]	27.8 [25.2, 30.4]

Note. CI: confidence interval; SE: standard error.

**Table 3:** Factors associated with comprehensive knowledge about HIV among women aged 15–49 years, 2000–2014: multivariate logistic regression analysis.

	Year 2000		Year 2006		Year 2011		Year 2014	
Independent Variable	aOR	95% CI	aOR	95% CI	aOR	95% CI	aOR	95% CI
Age group (years)								
<20	1		1		1		1	
20–34	1.0	[0.8, 1.2]	1.1	[0.9, 1.1]	1.2 ^*^	[1.1, 1.4]	1.2 ^*^	[1.1, 1.4]
35–49	1.1	[0.9, 1.4]	0.9	[0.7, 1.1]	0.9	[0.7, 1.1]	0.9	[0.8, 1.2]
Marital status								
Single	1		1		1		1	
Currently married	1.1	[0.9, 1.3]	0.8 ^***^	[0.6, 0.9]	1.0	[0.9, 1.2]	1.0	[0.9, 1.1]
Area								
Rural	1		1		1		1	
Urban	1.3 ^***^	[1.2, 1.5]	1.4 ^***^	[1.2, 1.6]	1.2 ^***^	[1.1, 1.4]	1.0	[0.9, 1.4]
Ethnicity								
Ethnic minority	1		1		1		1	
Kinh/Hoa	1.2	[0.9, 1.5]	0.8 ^*^	[0.7, 0.9]	1.2 ^*^	[1.1, 1.4]	1.2	[0.9, 1.4]
Education								
Primary or less	1		1		1		1	
Lower secondary	2.1 ^***^	[1.8, 2.5]	1.5 ^***^	[1.3, 1.7]	2.0 ^***^	[1.8, 2.3]	2.0 ^***^	[1.9, 2.7]
Upper secondary and tertiary	4.7 ^***^	[3.9, 5.6]	2.3 ^***^	[2.0, 2.7]	4.3 ^***^	[3.7, 5.0]	4.4 ^***^	[4.6, 6.5]
Socioeconomic status (quintile)								
Poorest 20%	1		1		1		1	
Near poor 20%	1.6 ^***^	[1.3, 2.1]	1.2 ^*^	[1.1, 1.5]	1.1	[0.9, 1.3]	1.4 ^***^	[1.2, 1.7]
Middle 20%	2.3 ^***^	[1.8, 3.0]	1.2 ^*^	[1.1, 1.5]	1.1	[0.9, 1.3]	1.4 ^***^	[1.2, 1.7]
Richer 20%	2.8 ^***^	[2.2, 3.6]	1.7 ^***^	[1.4, 2.1]	1.2	[0.9, 1.4]	1.8 ^***^	[1.5, 2.2]
Richest 20%	3.1 ^***^	[2.4, 4.0]	2.4 ^***^	[2.0, 3.0]	1.8 ^***^	[1.7, 2.2]	2.5 ^***^	[2.0, 3.2]
Ever tested for HIV								
No	1		1		1		1	
Yes	0.9	[0.8, 1.2]	1.9 ^***^	[1.6, 2.4]	1.9 ^***^	[1.6, 2.1]	2.0 ^***^	[1.7, 2.3]
Knows where to be tested for HIV								
No	1		1		1		1	
Yes	1.0	[0.9, 1.2]	2.3 ^***^	[2.1, 2.6]	1.9 ^***^	[1.7, 2.1]	2.0 ^***^	[1.8, 2.3]

Note. aOR: adjusted odds ratio; CI: confidence interval; ^*, ***^: significant at 0.05 and 0.001, respectively.

## Data Sharing Statement

The data that supports the findings of this study are available from http://mics.unicef.org/.
